# 210. Neutropenia and Associated Infectious Complications Among Kidney Transplant Recipients Receiving Valganciclovir Prophylaxis in the United States: An Administrative Claims Database Study

**DOI:** 10.1093/ofid/ofac492.287

**Published:** 2022-12-15

**Authors:** Vladimir Turzhitsky, Amit D Raval, Pamela Moise, Sanjay Merchant

**Affiliations:** Merck & Co., Inc., Boston, Massachusetts; Merck & Co., Inc., Boston, Massachusetts; Merck & Co., Inc., Boston, Massachusetts; Merck & Co., Inc., Boston, Massachusetts

## Abstract

**Background:**

Valganciclovir (VGCV) prophylaxis is commonly utilized to prevent cytomegalovirus (CMV) infection among high (D+/R-) and intermediate (R+) risk kidney transplant recipients (KTRs). Due to the myelosuppressive effects of VGCV, KTRs may be at an increased risk of opportunistic infections (OIs) resulting in interruptions to VGCV therapy. Therefore, we aimed to quantify the clinical burden of neutropenia among adult KTRs receiving VGCV prophylaxis.

**Methods:**

A retrospective cohort design was utilized to identify adult KTRs who received VGCV prophylaxis using the IBM MarketScan Commercial and Medicare Supplemental claims databases. First-time KTRs between 1/1/2012 - 12/31/2018 with 1 year of continuous pharmacy and medical coverage before (baseline period) and after (follow-up period) were included. KTRs with a History of other solid organ transplantation were excluded. Fills of ≥ 1 prescription of VGCV (either 450 mg or 900 mg/day) within 30 days of the kidney transplant (KT). Neutropenia events were identified as either ≥ 1 inpatient, or ≥ 2 outpatient ICD-9/ICD-10 diagnoses codes within 14 days.

**Results:**

Of the 4,965 adults KTRs with baseline and follow-up enrollment, 3,258 (66%) used VGCV prophylaxis. 311/3256 (9.5%) developed neutropenia within 1-year post KT. Baseline characteristics were similar between those with and without neutropenia (table 1). The majority received the following pre-transplant immunosuppression: tacrolimus (78.9%), mycophenolate mofetil (62.8%) and steroids (prednisone, 79.3%, methylprednisolone 10.6%). KTRs with neutropenia had a nearly two-fold increase in the development of CMV infection compared to those without neutropenia. In addition, KTRs with neutropenia had significantly higher rate of bacterial or fungal infections compared to those without neutropenia (Table 2). Interruptions in VGCV prophylaxis (gap >15 days) were more common in KTRs with neutropenia (P< 0.001).

**Figure:**
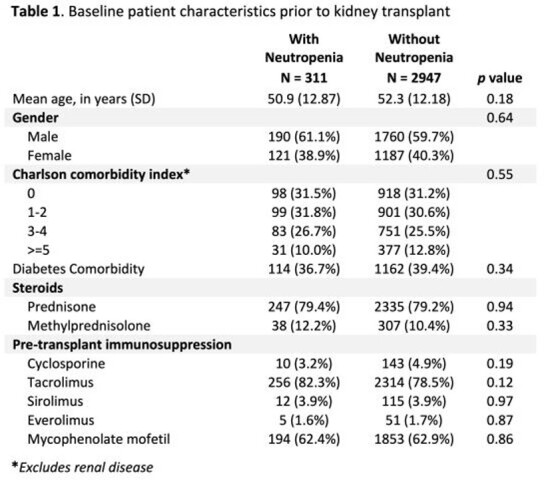

**Figure:**
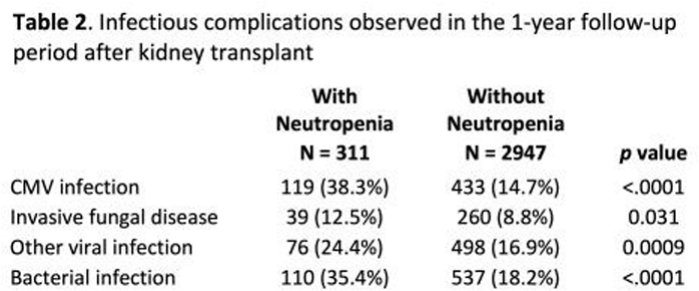

**Conclusion:**

Our findings showed that neutropenia was associated viral, bacterial, and fungal OIs among adults KT receiving VGCV prophylaxis. The findings highlight the needs for interventions to reduce neutropenia and its associated OIs. These results suggest that clinical management steps to reduce rates of neutropenia among KT recipients may warrant further study.

**Disclosures:**

**Vladimir Turzhitsky, PhD**, Merck & Co., Inc.,: Employee|Merck & Co., Inc.,: Stocks/Bonds **Amit D. Raval, PhD**, Merck and Co., Inc.: Employee of Merck|Merck and Co., Inc.: Stocks/Bonds **Pamela Moise, PharmD**, Merck & Co., Inc: Employee & Shareholder **Sanjay Merchant, PhD**, Merck & Co., Inc.: Stocks/Bonds.

